# Verotoxin A Subunit Protects Lymphocytes and T Cell Lines against X4 HIV Infection *in Vitro*

**DOI:** 10.3390/toxins4121517

**Published:** 2012-12-14

**Authors:** Pei Lin Shi, Beth Binnington, Darinka Sakac, Yulia Katsman, Stephanie Ramkumar, Jean Gariepy, Minji Kim, Donald R. Branch, Clifford Lingwood

**Affiliations:** 1 Department of Biochemistry, University of Toronto, Ontario M5G 1X8, Canada; E-Mail: peilinss@yahoo.ca; 2 Division of Molecular Structure and Function and Research Institute, The Hospital for Sick Children, Ontario M5G 1X8, Canada; E-Mail: bbinn@sickkids.ca; 3 Canadian Blood Services, Toronto, Ontario M5G 2M1, Canada; E-Mails: darinka.sakac@blood.ca (D.S.); author-yulia.katsman@utoronto.ca (Y.K.); 4 Laboratory Medicine & Pathology, University of Toronto, Ontario M5G 1X8, Canada; E-Mails: steph.ramkumar@utoronto.ca (S.R.); mminji.kim@mail.utoronto.ca (M.K.); donald.branch@blood.ca (D.R.B.); 5 Department of Medical Biophysics & Pharmaceutical Sciences, University of Toronto, Ontario M5G 1X8, Canada; E-Mail: gariepy@sri.utoronto.ca; 6 Sunnybrook Research Institute, Sunnybrook Health Science Centre, Toronto M4N 3M5, Canada; 7 Department of Medicine, University of Toronto, Ontario M5G 1X8, Canada; 8 Division of Cell and Molecular Biology, Toronto General Research Institute of the University Health Network, Toronto, Ontario M5G 2M9, Canada

**Keywords:** verotoxin, HIV, AIDS, PBMCs, anergy

## Abstract

Our previous genetic, pharmacological and analogue protection studies identified the glycosphingolipid, Gb_3_ (globotriaosylceramide, P^k^ blood group antigen) as a natural resistance factor for HIV infection. Gb_3_ is a B cell marker (CD77), but a fraction of activated peripheral blood mononuclear cells (PBMCs) can also express Gb_3_. Activated PBMCs predominantly comprise CD4^+^ T-cells, the primary HIV infection target. Gb_3_ is the sole receptor for *Escherichia coli* verotoxins (VTs, Shiga toxins). VT1 contains a ribosome inactivating A subunit (VT1A) non-covalently associated with five smaller receptor-binding B subunits. The effect of VT on PHA/IL2-activated PBMC HIV susceptibility was determined. Following VT1 (or VT2) PBMC treatment during IL2/PHA activation, the small Gb_3_^+^/CD4^+^ T-cell subset was eliminated but, surprisingly, remaining CD4^+^ T-cell HIV-1_IIIB_ (and HIV-1_Ba-L_) susceptibility was significantly reduced. The Gb_3_^-^Jurkat T-cell line was similarly protected by brief VT exposure prior to HIV-1_IIIB_ infection. The efficacy of the VT1A subunit alone confirmed receptor independent protection. VT1 showed no binding or obvious Jurkat cell/PBMC effect. Protective VT1 concentrations reduced PBMC (but not Jurkat cell) proliferation by 50%. This may relate to the mechanism of action since HIV replication requires primary T-cell proliferation. Microarray analysis of VT1A-treated PBMCs indicated up regulation of 30 genes. Three of the top four were histone genes, suggesting HIV protection via reduced gene activation. VT blocked HDAC inhibitor enhancement of HIV infection, consistent with a histone-mediated mechanism. We speculate that VT1A may provide a benign approach to reduction of (X4 or R5) HIV cell susceptibility.

## 1. Introduction

Verotoxin or Shiga-like toxins are a family of AB_5_ subunit toxins produced by enterohemorrhagic *E. coli* (EHEC)*.* Gastrointestinal infection with VT producing EHEC is the primary cause of hemolytic uremic syndrome [1]. VT1 and VT2 are 60% homologous but VT2 is associated with more severe clinical disease [2]. VTs belongs to a group of ribosomal inactivating proteins (RIPs) that inhibit protein synthesis in target cells by specifically removing an adenine residue in the 28S rRNA via its *N*-glycanase activity [3,4]. VTs bind, via their pentameric B subunit array, to their receptor GSL, Gb_3_ which alone [5] mediates their internalization and retrograde transport to the Golgi and then ER, where the A subunit separates from the holotoxin to be translocated into the cytosol to inhibit protein synthesis and kill the cell [6]. However, without the receptor binding B subunits, the A subunit is non-toxic. Several RIPs have been shown to have anti viral activity [7] but the exact mechanism is not defined. 

Ruminant animals naturally harbor EHEC in their digestive system without any harmful effect [8] and benefit from their anti-viral effect. The enzymatic A subunit of VT has been shown to inhibit expression and replication of two bovine retroviruses, bovine leukemia virus (BLV) and bovine immunodeficiency virus (BIV) [9,10,11]. A major characteristic of in vivo BLV infection in bovine PBMCs is spontaneous lymphocyte proliferation *in vitro*. VT1 treatment has been shown to inhibit this process without any cytotoxic effect or altered response to normal immune stimulants [9]. The catalytic activity of the A subunit is required for the inhibition effect since the catalytically inactive mutant VT1A, E167D, was ineffective. Furthermore, the BLV p24 core protein expression was reduced by VT1A treatment. However, this reduction was only seen in the cell-associated fraction and not in the culture supernatant, which suggests VT1A specifically eliminates BLV infected cells by lysis [12]. Studies with BIV also suggest VT specifically inhibits viral production by inducing apoptosis in infected cells [11]. However, no direct binding of VT to bovine PBMCs or BLV has been detected [11]. Since BLV infected bovine B cells showed greatly increased uptake of macromolecules < 70kDa, the specificity of VT to infected cells is thought to be a result of increased cell membrane permeability caused by viral infection [12]. 

Human CD4^+^ T-cells contain a small subpopulation which also express Gb_3_ [13,14] and Gb_3_ has been defined as a natural resistance factor against HIV infection [15,16]. The effect of verotoxin on PBMC HIV infection was therefore investigated. 

## 2. Results

### 2.1. PBMC Antigen Expression

PHA/IL-2-activated PBMCs are over 95% T-cell blasts [17]. This was confirmed using flow cytometry for the PBMC cell cultures used in this study. Freshly isolated PBMCs were activated with PHA/IL-2 for 4 days. Cells were then stained with anti-CD3, anti-CD14 or anti-CD19 antibody for detection of T-cells, monocytes and B cells respectively by FACS. The results (Table 1) showed that over 97% of the cell population was T-cells. Thus, the vast majority of the PBMC cell culture is susceptible to the T-cell-tropic HIV-1_IIIB_ virus. The PBMC cell surface staining was not affected by VT treatment (Table 1). Although only a small percentage (<2%) of CD4^+^ T-cells co express Gb_3_ [14] we nevertheless, determined the effect of VT on activated PBMCs and their susceptibility to HIV-1 infection. 

**Table 1:** Fluorescent-activated cell sorting (FACS) analysis of peripheral blood mononuclear cell (PBMC) lymphoid antigen expression.

**(a) Markers of PHA/IL-2 activated PBMC lymphoid subsets**					
**Antigen**			**% positive**		
CD3-T cells			97.4		
CD14-monocytes			2.0		
CD19-B cells			1.5		
**(b) Cell surface marker labeling of VT treated PHA/IL-2 activated PBMCs**					
**Treatment**			**%CD3+ve**		
Untreated			97.4		
1 µg/mL VT1			95.7		
1 µg/mL VT1A			94.2		
1 µg/mL VT1B			97.9		
**(c) Effect of VT1A treatment on CD4 positive vs. CD8 positive T cell composition**					
**Treatment**	% CD3^+^ T cells *	% CD4^+ ^CD3^+^ T cells *		% CD8^+^ CD3^+ ^T cells *	CD4^+^ CD8^+^ T cells *
Untreated Control	85.1	41.7		47.0	0.9
1 µg/mL VT1A	88.1	43.5		49.2	0.9

* Different T cell antigen markers.

### 2.2. Metabolic Labeling of PBMC GSLs

**Figure 1:**
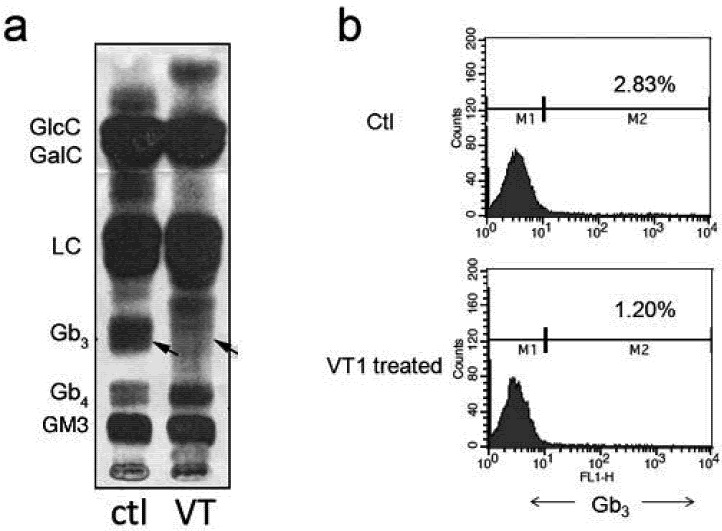
Gb_3_ expression in PHA/IL-2 activated PBMCs. (**a**) PBMCs were treated with 500 ng/mL of Verotoxin-1 (VT1) and activated with PHA/IL-2. On day 2, 2 µCi/mL of ^14^C-galactose and 0.5 µCi/mL of ^14^C-serine were added to the cell culture overnight. The cells were then washed once with phosphate-buffered saline (PBS), subjected to total neutral GSL extraction and purified by silica chromatography. GSLs were resolved using TLC and detected by autoradiography. Position of GSL standards, glucosyl ceramide, galactosyl ceramide, lactosyl ceramide, Gb_3_, Gb_4_ and GM3 ganglioside, are shown on the left. Lane 1—control untreated PBMCs, lane 2—VT1 treated PBMCs. Arrows indicate Gb_3_; (**b**) PBMCs were activated and treated ± VT1 then analyzed for Gb_3_ expression by FACS staining with Alexa-488-VT1B on day 4. Plots represent Gb_3_ expression profiles. Negative gate was set using unlabeled control.

Gb_3_ is the only known receptor for VT, which can trigger the receptor-mediated retrograde transport pathway that results in ribosomal inactivation and cell death. The effect on Gb_3_ synthesis by activated PBMCs was first determined using ^14^C-galactose/serine metabolic labeling. Activated PBMCs were treated with or without VT1 and then metabolically labeled with ^14^C-galactose/serine for 18 h. GSLs were extracted, separated by TLC and detected by autoradiography (Figure 1a). Gb_3_ was detected as a minor species, which was lost in the VT treated sample, suggesting VT deleted the Gb_3_-expressing subpopulation in activated PBMCs. Gb_3_ staining was reduced from 2.8 to 1.2% (Figure 1b). This finding is in agreement with previous results, which showed PHA/IL-2 activation induced Gb_3_ expression in PBMCs [13], but that only a very small fraction of CD4^+^ T-cells co express Gb_3_ [14]. Interestingly, although Gb_3_ was eliminated, Gb_4_ was increased in the VT treated PBMCs (Figure 1b). 

These results are consistent with a small, VT-sensitive population within activated PBMCs. VT elimination of this small population would be unlikely to have significant effect on HIV-1 susceptibility of activated PBMCs; unless, to explain the natural resistance provided by Gb_3_ this would prove to be a key regulatory cell type. 

### 2.3. Effect of VT on HIV PBMC Infection

**Figure 2:**
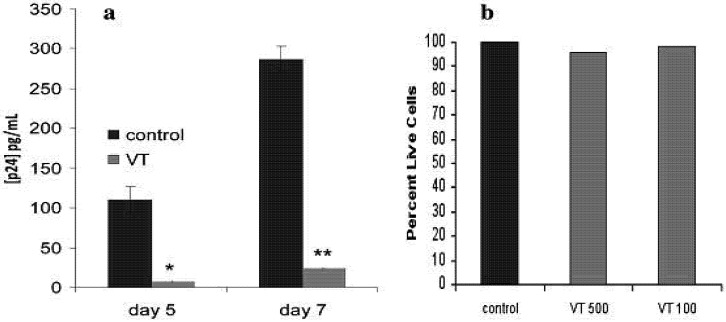
Effect of VT1 on PBMC susceptibility to HIV infection. Panel (**a**) PBMCs were treated with 500 ng/mL of VT1 and activated with PHA/IL-2 for 3 days. PBMCs were infected with HIV-1_IIIB_ for 1 h, washed and measured by p24^gag^ ELISA after 5 and 7 days. >90% inhibition was observed. Asterisks indicate statistical significance compared to control (* p < 0.05; ** p < 0.03); Panel (**b**) Cell viability for PBMCs activated in the presence of 500 or 100 ng/mL VT1 was monitored by trypan blue dye exclusion.

The effect of VT treatment of PBMCs on HIV susceptibility was unexpected since the HIV target cell population was unaffected by VT1 treatment (Table 1). VT1 treated PBMCs became highly refractory to HIV infection (Figure 2a). Consistent with a minor Gb_3_ expressing fraction, the viability of VT treated and control PBMCs was equivalent (Figure 2b).

### 2.4. Comparison of VT Subunits for Protection of PBMCs against HIV Infection

**Figure 3:**
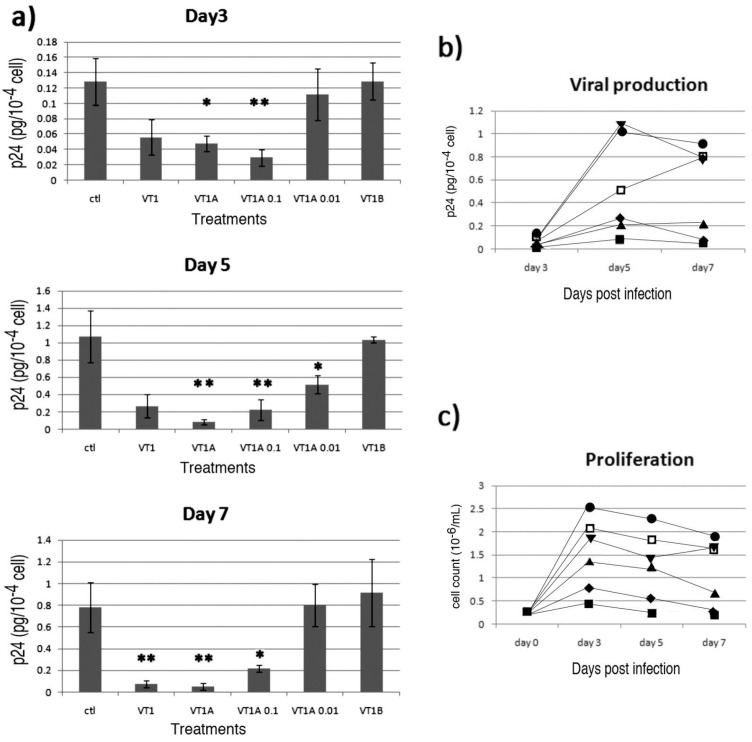
Verotoxin-1 A subunit protects PHA/IL-2 activated PBMCs against HIV-1_IIIB_ infection. PBMCs were treated with VT1 (1 µg/mL), VT1A (1, 0.1, 0.01 µg/mL) or VT1B (1 µg/mL) during PHA/IL2 activation. HIV-1_IIIB _infection was conducted 4 days post activation (m.o.i. = 0.3, *n* = 4). (**a**) Viral production was measured by p24^gag^ ELISA for day 3, 5 and 7 of infection. Asterisks indicate statistical significance compared to control (* p < 0.05; ** p < 0.03); (**b**) Viral production over time. All viral production values were normalized by cell count; (**c**) Viable cell count over time by Trypan blue exclusion. (▼ = control, ♦ = 1 µg/mL VT1, ■ = 1 µg/mLVT1A, ▲ = 0.1 µg/mL VT1A, □ = 0.01 µg/mL VT1A, ● = 1 µg/mL VT1B). A subunit was as effective as holotoxin to induce HIV resistance.

To determine whether the VT1 inhibition of HIV infection was receptor independent, we compared the effect of VT1 with that of the separated VT1A and VT1B subunits. We found that at 1 µg/mL VT1A subunit was as effective as VT1 holotoxin, and that the receptor binding VT1B subunit pentamer was not protective (Figure 3). VT1A subunit protection was dose dependent, showing inhibition of infection at 100 but not 10 ng/mL.

VT1 and protective concentrations of VT1A reduced the proliferation of activated PBMCs (Figure 3c). The HIV infection monitored by p24^gag^ is normalized to the cell number, but the correlation with efficacy implicates reduced cell growth in the mechanism of action. 

**Figure 4:**
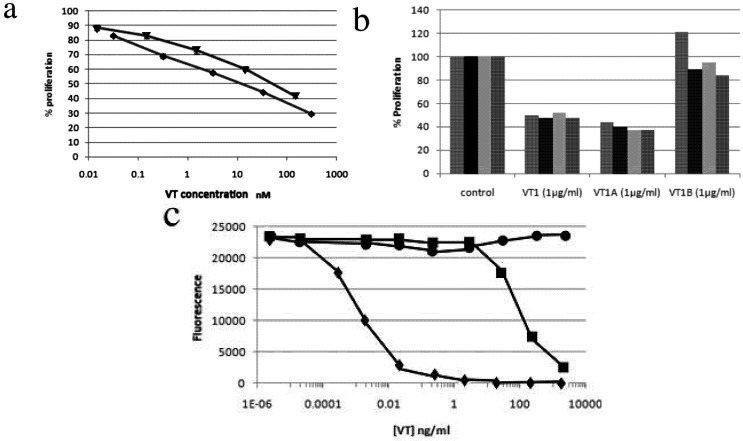
PBMC proliferation after PHA/IL-2 activation and VT or VT subunit treatment. VT was added to lymphocytes after isolation and remained present during PHA/IL-2 activation. Cell proliferation was measured on day 4 using alamarBlue^®^ fluorescent dye indicator assay. (**a**) VT1 (▼), VT1A (♦) titration of percentage proliferation compared to no VT control; (**b**) PBMC samples were collected from 4 different donors and activated in the presence of 1 µg/mL VT1, VT1A or VT1B. Cell number was determined on day 4. (**c**) Cytotoxcity titration curve using VT sensitive Gb_3_^+^ THP-1 monocytic cell line: VT1 (♦), VT1A (■) or VT1B (●). The toxicity of the VT1A indicates a maximum holotoxin contamination of 1/50,000.

The reduced PBMC proliferation during PHA/IL2 activation was highly consistent and seen for VT1 and VT1A but not VT1B subunits (Figure 4). The dose response for PBMC was quite different from the toxicity observed for Gb_3_ expressing cells (Figure 4a *cf.* c). Although little effect of VT1A on PBMC protein synthesis in the short-term could be detected, treatment for 4 days reduced ^3^H-leucine incorporation into TCA insoluble material by 50% (not shown). No effect of VT1A on global phosphotyrosine content of activated PBMCs was seen (not shown).

Since we found the verotoxin receptor glycosphingolipid, Gb_3_, to be a resistance factor for HIV infection in vitro [14,15], we expected that treatment of activated PBMCs with VT1 would eliminate the small fraction of Gb_3_ expressing cells we have shown to be present and thereby increase susceptibility to subsequent HIV infection. Although this subpopulation was effectively removed by VT1 treatment, the VT1 (or VT2, not shown) treated cells became highly refractory to HIV infection and this was found to be a Gb_3_/B subunit independent, VT1A subunit-mediated event. Even though the VT1A subunit was removed by cell washing, the activated PBMCs remained resistant to infection. 

### 2.5. VT also Inhibits Jurkat T-Cell Infection by HIV-1

**Figure 5:**
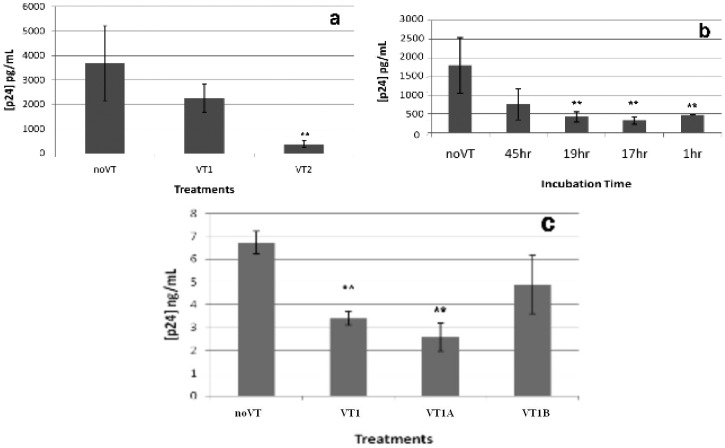
VT treatments of Jurkat-C cells significantly reduce subsequent HIV-1_IIIB _infection. (**a**) JKT-C cells were treated for 3 days with VT1 or VT2 (1 µg/mL) and the toxins were removed by washing with culture media prior to infection with HIV-1_IIIB_ (m.o.i. = 0.1). Post infection supernatants were collected at day 7 and HIV-1 viral production was measued by p24^gag^ ELISA (**b**) A time course was conducted to determine the minimum preincubation time required for VT1 treatment to achieve viral reduction. p24 measured on day 6. Error bar represents standard error mean (*n* = 4). One hour pretreatment was sufficient.

**Figure 6:**
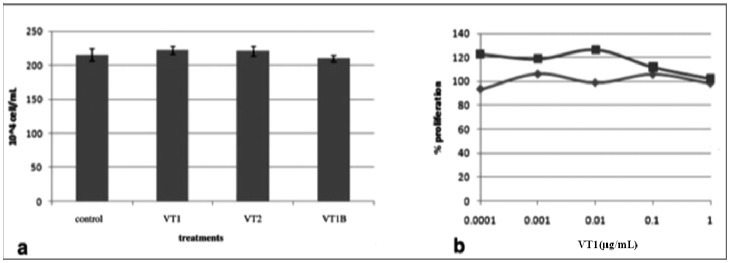
Verotoxin is non-toxic to Jurkat-C cells. Cells were treated with different concentrations of VT1 or VT1B in 10× serial dilutions. Cell proliferation was measured by the redox dye alamarBlue^®^. (**a**) JKT-C cell viability with 1 μg/mL VT1, VT2, or VT1B treatment for 3 days was measured by Trypan Blue dye exclusion assay. Error bars represent standard error mean (*n* = 4); (**b**) JKT-C cells were treated with VT1B (■) or VT1 (♦) for 3 days and proliferation was calculated as percentage of no VT control. One of three similar experiments is shown.

The Gb_3_-negative Jurkat-C human T-cell line is a standard surrogate for primary human T-lymphocyte HIV infection. VT is effective at reducing HIV Jurkat cell infection (Figure 5). As for PBMCs, VT1A subunit was as effective as holotoxin (not shown). Initially cells were preincubated with VT as for the PBMCs (Figure 5a) but this prolonged preincubation proved unnecessary (Figure 5b). An hour preincubation was sufficient for optimal inhibition. Unlike for PBMCs, VT had no effect on Jurkat cell proliferation or viability (Figure 6).

### 2.6. Gene Expression Array Analysis of VT1A Treated PHA/IL-2-Activated PBMCs

To further define the effects of VT1A treatment on normal function of PBMCs, gene expression array analysis was conducted. Isolated PBMCs were activated with PHA/IL-2 for 4 days. Test samples were treated with 1 µg/mL of VT1A at the time of activation and control samples were treated with vehicle only (*n* = 3). Total RNA was extracted using TRIzol^®^ reagent and sent for gene expression array analysis using Human WG-6 Expression BeadChip. Array results were analyzed using the LIMMA algorithm. Genes with an adjusted p value of <0.03 were considered differentially expressed. Table 2 shows the gene changes with *p* value of <0.1. Overall, the effect of VT1A on PBMC gene expression was highly specific since only 49 genes were significantly affected out of a total of 36,604 genes. 30 genes were up-regulated (0.082%) and 19 genes were down-regulated (0.051%) (Table 2). The most striking finding was that 10 of the upregulated genes (three of the top four) belonged to histone cluster proteins.

**Table 2:** Differentially expressed genes of VT1A treated PBMCs *vs.* control PBMCs. Only genes with adjusted *p* value of less than 0.1 are listed. Fold change represents expression level difference between VT1A treated samples vs. control no VT samples. Positive values indicate an increase and negative values indicate a decrease.

Symbol	Definition	Fold Change	adj. *p* value
**HIST1H4B**	histone cluster 1, H4b	5.22	0.000185
**HIST1H4H**	histone cluster 1, H4h	5.01	0.000192
SPP1	secreted phosphoprotein 1, transcript variant 2	−4.50	0.000192
**HIST1H4F**	histone cluster 1, H4f	3.95	0.000363
INDO	indoleamine-pyrrole 2,3 dioxygenase	−3.84	0.000466
CSF2	colony stimulating factor 2 (granulocyte-macrophage)	3.63	0.000515
SPP1	secreted phosphoprotein 1, transcript variant 1.	−3.94	0.000515
LYZ	lysozyme (renal amyloidosis)	−3.51	0.000547
GADD45B	growth arrest and DNA-damage-inducible, beta	3.43	0.000547
CCL24	chemokine (C-C motif) ligand 24	−3.45	0.000547
IL9	interleukin 9	3.47	0.000699
IL9	interleukin 9	3.17	0.00112
TM4SF19	transmembrane 4 L six family member 19	−3.11	0.00112
IL8	interleukin 8	−3.20	0.00151
SLC11A1	solute carrier family 11 (proton-coupled divalent metal ion transporters), member 1.	−3.33	0.00154
IL1B	interleukin 1, beta	−2.78	0.00317
MMP9	matrix metallopeptidase 9	−2.73	0.00358
**HIST2H4A**	histone cluster 2, H4a	2.71	0.00569
TAC1	tachykinin, precursor 1, transcript variant alpha	2.62	0.00627
PPP1R15A	protein phosphatase 1, regulatory (inhibitor) subunit 15A	2.60	0.00627
TYROBP	TYRO protein tyrosine kinase binding protein, transcript variant 1.	−2.80	0.00627
GNLY	granulysin, transcript variant NKG5	−2.52	0.00733
OSM	oncostatin M	2.49	0.00758
TM4SF19	PREDICTED: transmembrane 4 L six family member 19, transcript variant 2	−2.60	0.00758
**HIST1H2AC**	histone cluster 1, H2ac	2.49	0.00972
OR8H2	olfactory receptor, family 8, subfamily H, member 2	2.51	0.00972
FOS	v-fos FBJ murine osteosarcoma viral oncogene homolog	2.58	0.972
TNFSF4	tumor necrosis factor (ligand) superfamily, member 4	2.35	0.0185
IL8	interleukin 8	−2.29	0.0185
FOSB	FBJ murine osteosarcoma viral oncogene homolog B	2.32	0.0185
**HIST1H2BF**	histone cluster 1, H2bf	2.34	0.0220
GNLY	granulysin, transcript variant 519	−2.24	0.0223

### 2.7. VT1 Blocks HDAC Inhibitor Effect on HIV Infection

**Figure 7:**
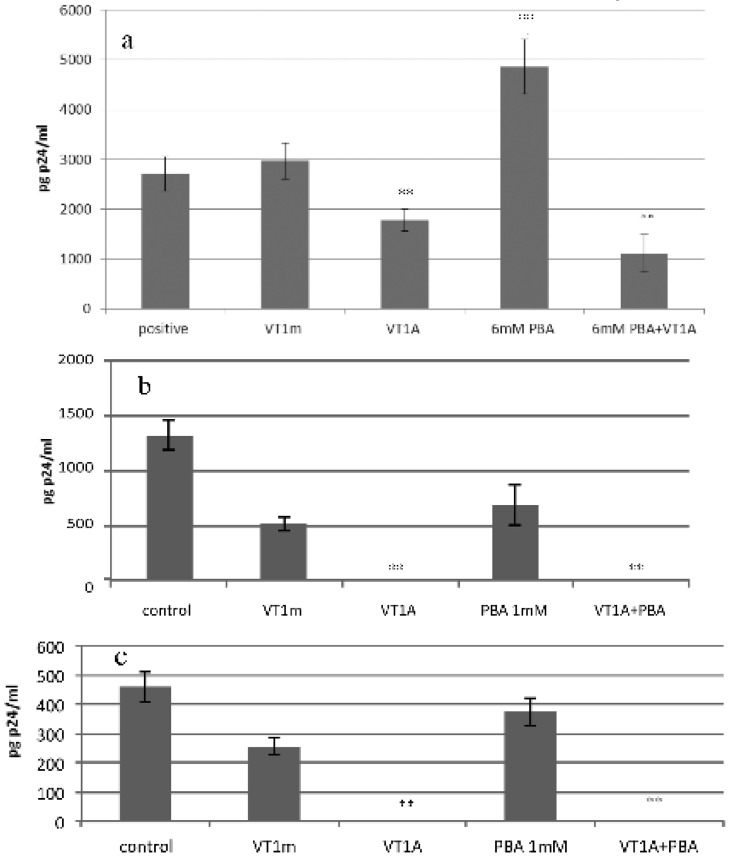
VT1A counters Histone deacetylase (HDAC) inhibitor effect on PBMC HIV susceptibility. PBMCs were PHA/IL2 activated in the presence of inactivated A subunit containing holotoxin (VT1m), VT1A , phenyl butyrate (PBA) or PBA + VT1A and tested for susceptibility to HIV infection (day 4). Panel (**a**) X4 HIV_IIIB_ and 6mM PBA, panel (**b**) X4 HIV_IIIB_ and 1mM PBA, panel (**c**) R5 HIV_Ba-L_ and 1mM PBA ** indicates *p* < 0.03 (*n* = 4).

Gene activation is required for HIV infection [18]. Histones regulate gene transcription via their acetylation status and histone deacetylase inhibitors have been probed as a means to activate transcription to detect and treat latent HIV provirus transcripts integrated within the target cell genome [19]. A VT1A subunit effect on histone acetylation could provide a basis for its protection against HIV infection. The effect of phenyl butyrate (PBA), a known HDAC inhibitor [20], on VT1 protection against HIV infection was tested (Figure 7). 

VT1, but not its A subunit inactivated mutant [21], reduced PBMC susceptibility to HIV, whereas PBA significantly increased infection. Combining VT1 with PBA resulted in a (even more) pronounced protection against infection. Either PBA reversal of VT1 protection or VT1 reversal of PBA enhancement of infection would serve to indicate a shared mechanism of action. VT1 blocking PBA increased HIV susceptibility supports a role for VT promotion of histone deacetylation to reduce gene activation and engender HIV resistance. At the 6 mM concentration used for HDAC inhibition, PBMC proliferation during PHA/IL2 activation was reduced. The experiment was therefore repeated at 1 mM PBA and tested against both X4 (Figure 7b) and R5 (Figure 7c) HIV. At 1mM PBA pretreatment, the stimulation of HIV infection was not maintained but the VT1A inhibition of both X4 and R5 HIV infection was clearly demonstrated.

### 2.8. Discussion

The verotoxin A subunit is an *N*-glycanase, removing an adenine base from position 4324 of the 28S RNA of the 60S ribosomal subunit [4] to inhibit protein synthesis and kill the cell. To achieve this, the A subunit must internalize [22], undergo proteolytic cleavage [23], traffic to the ER [24] and translocate into the cytosol [6]. The A subunit can have additional effects, including increasing the half-life of selected mRNAs [25,26] and DNA can be a substrate for the *N*-glycanase activity [27]. The A subunit can induce apoptosis [28]. However in all these cases, A subunit cytosolic access is dependent on B subunit mediated cell binding and intracellular traffic. A subunit antiviral activity has been reported [11] but prior cell infection was required to increase cell permeability to allow cell entry [28]. In contrast, our studies indicate the A subunit can interact with uninfected cells to induce a state of anergy, resistant to HIV infection. 

The A subunit contains hydrophobic residues which can mediate cell interaction [29]. However, we were unable to detect specific VT1A binding to PBMCs and fluid phase micro pinocytosis is the most likely mechanism of VT1A cell entry, but the mechanism for cytosolic access if required, is not obvious. The mechanism of A subunit cell targeting and internalization remains to be determined. Ribosome inactivating proteins have been shown to have anti-HIV activity [7]. These include bacterial toxins [16]. Depurination of HIV-1 RNA long terminal repeats may hinder HIV integration [30]. However, RIPs can also activate the host MAP kinase pathway to counter this and increase infectivity [31]. VT has been shown to activate this pathway [32]. RIP-immunoconjugates have been promoted for HIV intervention [33].

In several animal species, verotoxin has been found to be protective against related retroviral infection [9,34] and the A subunit is toxic to bovine retrovirus infected cells [11], although binding to such cells could not be demonstrated. In these cells, increased permeability of the infected cells was proposed as the selectivity basis. 

In the present studies however, the VTA subunit is added to the target human lymphocytes and removed before any HIV infection. There is no change in viability as defined by vital dye exclusion, but growth rate is reduced. Inhibition of “spontaneous lymphocyte proliferation” by VT1A in the bovine was reported [9]. In our studies, PBMC proliferation during activation was clear, though reduced by 50%. PBMC proliferation post HIV infection was <10% control. This inhibition correlated with, but was not proportional to, the degree of inhibition of PBMC proliferation. However, we saw no effect on Jurkat T cell proliferation, so growth inhibition may be downstream of the primary VT effect only in primary lymphocytes. VT treatment during lymphocyte activation induces a form of subsequent T cell anergy. This resistance is effective against both X4 and R5 HIV infection. VT1 increased histones, indicated from our mRNA microarray data, could increase chromatin to decrease gene activation and hence proliferation, to provide the basis of the induced prolonged HIV resistance. HDAC inhibitors are used to facilitate transcription to allow detection of latent HIV virions integrated in the genome [19]. The VT blockade of HDAC inhibitor enhanced HIV infection we observed, is consistent with histone mediated VT1A protection against HIV. The PBA HDAC inhibitor reduced PBMC proliferation during activation, indicating T cell growth reduction *per se* can be insufficient for HIV resistance.

VT1A reduced T cell division might counteract the excessive T cell proliferation characteristic of early HIV infection [35] and reduced T lymphocyte proliferation might prove a small price to pay for prophylactic HIV resistance.

## 3. Experimental Section

### 3.1. Cell Lines

Acute T-cell leukemia-derived Jurkat-FHCRC cells (JKT-C) were obtained from Dr. D. Branch. The human acute monocytic leukemia cells (THP-1) were obtained from NIH AIDS Research and Reference Reagent Program (Rockville, MD, USA). Both cell lines were cultured in complete RPMI1640 medium (Wisent Inc, St-Bruno, QC, USA) supplemented with 10% fetal bovine serum (FBS) (Sigma-Aldrich, Oakville, ON, USA), 100 IU penicillin and 100 µg/mL streptomycin (Wisent Inc., Quebec, Canada) at 37 °C in 5% CO_2_. 

### 3.2. Isolation and Activation of Peripheral Blood Mononuclear Cells (PBMCs)

PBMCs were prepared as previously described [36]. Briefly, fresh whole blood was collected from healthy donors following informed consent in acid citrate dextrose (ACD). The blood was mixed in a 1:1 ratio with complete RPMI1640 medium and overlaid on Ficoll-Paque PLUS (GE Healthcare AB, Stockholm, Sweden) and centrifuged at 1800 rpm for 45 min. The middle PBMCs layer was washed three times with Dulbecco’s PBS without MgCl_2_ and CaCl_2_. Viable PBMCs were re-suspended in complete medium at ~1 × 10^6^ cells per mL. Phytohemagglutinin (PHA) and interleukin-2 (IL2) were added to freshly isolated PBMCs to a final concentration of 5 µg/mL and 100 U/mL respectively and cells cultured at 37 °C in 5% CO_2_ for 4 days. 

### 3.3. HIV Infection

HIV-1_IIIB_ and R5 HIV-1_Ba-L_ were from the National Institutes of Health AIDS Research and Reference Reagent Program, Division of AIDS, National Institute of Allergy and Infectious Diseases. In the Level 3 containment facility at the University of Toronto, HIV-1_IIIB_ viral stocks were grown in JKT-C cells, and multiplicity of infection (m.o.i.) was determined as described using MT-4 cells [13]. HIV-1_Ba-L_ viral stocks were grown in PBMCs, and infectious dose calculated from total p24^gag^levels [16]. Infection of cells was as previously described [13,16]. Briefly, 5 × 10^5^ cells were incubated with HIV-1 (m.o.i. = 0.1) for 1 h at 37 °C, the cells washed three times with phosphate-buffered saline (PBS), and cultured in complete medium (containing IL-2 (100 U/mL) for PBMCs). Culture supernatant aliquots were taken over time to determine viral production by ELISA to measure p24^gag^antigen levels.

### 3.4. Preparation of VT1 and VT2

Verotoxin-1 (VT1) and Verotoxin-2 (VT2) were purified as described [37]. VT1B subunit was purified from *E coli* strain JB120 [38]. Recombinant VT1A subunit was obtained by HPLC separation of the denatured toxin [39]. Alexa 488-VT1B was prepared in our lab using Alexafluor-488 tetrafluorophenyl (TFP) ester (Invitrogen, Burlington, ON, Canada) according to product manual. 

### 3.5. Cytotoxicity Assay

Target cells ~ 10^5^ cells in 200 µL were dispensed into microtiter plate wells. 200 µL VT1, VT2, VT1A or VT1B solution was added in 10-fold serial dilutions. Cells were incubated for 4 days at 37 °C in 5% CO_2_. On day 4, cell proliferation was quantified using alamarBlue^®^ assay. The fluorescence was measured using 540 nm excitation wavelength and read at 590 nm emission wavelength using Spectra MAX plate reader, Gemini EM (Molecular Devices, Toronto, ON, Canada)**.** Proliferation was calculated as a percentage in comparison to no VT control (100%).

### 3.6. Protein Synthesis

^3^H-leucine incorporation was used to assess VT effect on nascent protein synthesis. ~6 × 10^5^ Cells were incubated in leucine-free DMEM media without serum (Specialty media, Phillipsburg, NJ, USA) for 3 h. Jurkat-C and THP-1 cells were treated with 1 µg/mL VT1, VT1A or no VT for 3 h. PBMCs were treated with 1 µg/mL VT1A for 4 days during activation or for 3 h on day 4 of activation. 5 µCi of ^3^H-leucine (Amersham Biosciences, Buckinghamshire, UK) were added for incorporation at 37 °C for 30 min. Cells were washed 3 times with PBS and 10% trichloroacetic acid was used to precipitate protein. The protein pellet was suspended in scintillation fluid. Incorporation was quantitated using a beta-counter (Beckman LS6500, Bioanalytical Systems Group, Mississauga, ON, USA). Alternatively cell pellets were lysed and resolved by 12% reducing SDS-PAGE gel, and proteins detected by autoradiography.

### 3.7. Flow Cytometry

Fluorescent-activated cell sorting (FACS) was used to detect cell surface receptors on PBMCs. Approximately 5 × 10^5^ cells were incubated in 100 μL of 10% mouse serum (Sigma-Aldrich, Oakville, ON, Canada) in FACS buffer (PBS, 2% FBS, 0.1% sodium Azide, 5 mM EDTA) at 4 °C for 30 min to block F_c_ receptors and non-specific binding. After spinning at 2000 rpm (Micromax, Buckinghamshire, UK) the cell pellets were then re-suspended in 100 μL of FACS buffer containing 1 μg of mouse anti-human CD3-FITC IgG2aκ, CD14-APC IgG2aκ or CD19-PE IgG1κ antibody and incubated at 4 °C for 30 min in the dark. FITC mouse IgG2aκ, PE mouse IgG1κ and APC mouse IgG2aκ were used as negative controls for gating. All antibodies above were purchased from BD Pharmingen, San Diego, CA, USA. Cells were then pelleted and washed once with fresh FACS buffer and then diluted in 500 μL of FACS buffer for data collection and analyses using a FACS Calibur Analyzer or Becton Dickinson LSRII (Flow cytometry facility, Toronto MarS Building) equipped with Cell Quest^®^ or FACSDeva^®^ 3.0 software. For CXCR4 staining a panel of three antibodies was used. Cells were blocked with 10% normal goat serum (Vector Laboratories Inc, Burlingame, CA, USA) then incubated with 0.5 μg ofmouse anti-human CXCR4 primary antibodies: 12G5 (Bioscience, San Diego, CA, USA), MAB 173 (NIH AIDS), or MAB171 (R&D systems, Burlington, ON, USA) in 100 μL FACS buffer. After washing once with FACS buffer, the cells were incubated with 0.3 μg of secondary goat anti-mouse-Alexa-488 antibody (Invitrogen, Burlington, ON, Canada) in 100 μL FACS buffer for 30 min at 4 °C in the dark. A sample stained with secondary antibody only was used as negative control. For Gb_3_ labeling, cells were directly labeled with 5 μg/mL VT1B-Alexa 488 for 30 min at 4 °C in the dark. 

### 3.8. RNA Extraction

Freshly isolated PBMCs were activated with PHA/IL-2 and grown either with 1 μg/mL VT1A or no VT as control for 4 days. Cells were collected for total RNA extraction using TRIzol^®^ reagent (Invitrogen, Burlington, ON, Canada). Approximately 5~10 × 10^6^ cells were lysed by repetitively pipetting with 1mL of TRIzol followed by 5 min incubation at RT. 0.2 mL of chloroform was added and the sample was shaken vigorously for 15 s. After 3 min of incubation at RT the sample was centrifuged at 11,000*g* for 10 min at 2 °C. The white interphase was collected and incubated with 0.5 mL isopropyl alcohol for 10 min at RT. The precipitated RNA was centrifuged as before, washed once with 75% ethanol and centrifuged at 7500*g* for 5 min at 4 °C. RNA was re-dissolved in 0.01% diethylpyrocarbonate at 55 °C for 20 min. Concentration was determined by measuring *A*_260_ and quality of RNA was assessed by having an *A*_260_/*A*_280_ ratio of ~2 [40]. Samples were stored at −80 °C until further analysis.

### 3.9. Human Gene Expression Array Analysis

Purified total RNA samples were submitted to The Centre for Applied Genomics (TCAG, The Hospital for Sick Children Research Institute) for human gene expression array analysis. Total RNA was treated and amplified using the Illlumina^®^ TotalPrep^TM^-96 RNA Amplification Kit (Ambion, Auston, TX, USA). Total RNA was first reverse transcribed into single stranded cDNA. Then the second DNA strand was synthesized and the double stranded cDNA was purified using magnetic beads. The dsDNA was transcribed into Biotin-labeled cRNA *in vitro*. Finally, the resulting cRNA was purified by capturing with RNA binding beads. 

Human WG-6 Expression BeadChip from Illumina^®^ (San Diego, CA, USA) was used as the array platform. The results were analyzed at the Statistical Analysis Core Facility (The Hospital for Sick Children Research Institute, Toronto, ON, USA) with the assistance of Dr. Pingzho Hu.

### 3.10. Statistics

For microarray analysis, background correction was done with BeadStudio software (Illumina). The quantile normalization method implemented in lumi R package was used to normalize the data quantiles [41]. Differentially expressed genes were identified using LIMMA (linear models for microarray data) [42]. Briefly, a linear model was fitted for each gene in the data, and then an empirical Bayes (EB) method was used to moderate the standard errors for estimating the moderated *t*-statistics for each gene, which reduced the standard errors towards a common value. The corresponding *p*-values for the *t*-statistics were adjusted using the multiple testing procedure. Differentially expressed genes were identified by having a fold change >2 and adjusted *p*-value < 0.03.

HIV-1 infection p24^gag^ ELISA data and VT cytotoxicity assays were represented as the mean of several separate experiments, where n values are indicated in the Figure legends. Error bars represent standard error of the mean (+/− SEM). A two-tailed student’s *t*-test was performed where appropriate and *p*-values less than 0.05 were considered significant (*) and *p*-values less than 0.03 were considered highly significant (**).

## 4. Conclusions

The ribosome inactivating A subunit of verotoxin induces resistance to subsequent X4/R5 HIV-1 exposure in primary human T-lymphocytes and the Jurkat T cell line, independent of the B subunit and Gb_3_ receptor binding of the holotoxin. Reduced primary T cell proliferation post infection correlates with inhibition of infection.
